# Determination of neonatal case-specific fatality rates in a tertiary health institution in North Central Nigeria

**DOI:** 10.1186/s12887-021-02778-x

**Published:** 2021-07-07

**Authors:** L. I. Audu, A. T. Otuneye, A. B. Mairami, M. Mukhtar-Yola, L. J. Mshelia

**Affiliations:** 1 Department of Paediatrics and Child Health, Barau Dikko Teaching Hospital, Kaduna State University, Kaduna, Nigeria; 2 Department of Paediatrics, National Hospital Abuja, Abuja, Nigeria

**Keywords:** Neonatal mortality, Case-specific fatality, Abuja, Nigeria

## Abstract

**Background:**

The current neonatal mortality rate in Nigeria (37/1000) is among the highest in the world and the major causes have consistently been reported as sepsis, perinatal asphyxia and prematurity. However, case-specific fatality which defines the risk of dying from these and other neonatal morbidities is rarely emphasized. Determination of case-specific fatality rates (CSFR) may inform a change in our current approach to neonatal care interventions which may eventually bring about the much-needed reduction in our neonatal mortality rate. Our aim was to determine the case-specific fatality rates for the common causes of mortality among hospitalized neonates at the National Hospital Abuja (NHA).

**Methods:**

Relevant demographic and clinical data on all neonates admitted into the NICU at the NHA over a period of 13 months (January 2017 to February 2018) were extracted from the Neonatal Registry database and analyzed using appropriate statistical methods with the SPSS version 20 software. The case-specific fatality rates were computed for the predominant morbidities in addition to determination of the neonatal mortality rates and associated risk factors.

**Results and conclusion:**

A total of 730 neonates were admitted, out of which 391 (53.6%) were females, 396 (54.5%) were inborn and 396 (54.2%) were term. The three most prevalent morbidities were prematurity 272(37.2%), neonatal Jaundice 208(28.4%) and perinatal asphyxia 91(12.5%) while the most common causes of mortality were prematurity 47/113(41.6%), congenital malformations 27/113(23.9%) and perinatal asphyxia 26/113(23%). Congenital malformations had the highest case-specific fatality 27/83(32.5%) followed by Perinatal Asphyxia 26/91(28.6%) and prematurity 47/272(20.7%). The mortality pattern differed between inborn and out born babies. Implications of these case-specific fatality rates for targeted interventions are discussed.

## Background

Neonatal sepsis, perinatal asphyxia and prematurity have remained the leading causes of neonatal deaths reported from different parts of (the) Nigeria [1–5]. This pattern is not limited to Nigeria, having been described in other resource poor countries in Africa and Asia [6]. Specific interventions targeted at these morbidities have been intensely implemented across the country with only modest impact on neonatal mortality. For instance, according to the Nigerian Demographic Health Survey 2018 report, Neonatal mortality had only dropped by 3/1000 from 42/1000 to 39/1000 over a period of 28 years (1990–2018) [7]. It is therefore imperative to advocate for research driven changes to our current neonatal intervention strategies.

The 3 most important causes of neonatal mortality; perinatal asphyxia (PA), neonatal sepsis (NNS) and prematurity constitute 80–85% of all neonatal deaths in Nigeria [8]. This important information gives the numerical contribution of these disorders to neonatal death as clearly depicted by Ramadurg et al. [9], but does not sufficiently address the risk or probability of death associated with each of these and other neonatal morbidities. A more appropriate measure of this risk is the *case-specific fatality rate* which defines ‘the proportion of death within a designated population of a specified disease [10]. This is an indication of both the severity of the disease and standard of interventions available for the treatment of specific diseases. Information obtained from this analysis is invaluable in planning appropriate and effective health interventions. For instance, diseases with high fatality rate are often life-threatening and would require high level expertise and advanced technology to positively alter their natural course. In low- and medium-income countries, this level of intervention is limited to a few specialized hospitals. It is therefore important to identify the most common neonatal illnesses with high case-specific fatality rates as a step towards designing and implementing targeted interventions that will include a comprehensive inter-hospital referral protocol, to reduce neonatal mortality in Nigeria.

Case-specific fatality rates are not routinely computed in most studies on neonatal morbidity and mortality even when data for this important index of neonatal care is available from such studies. Where this neonatal index is computed, it may not be so designated [3] or its relevance may not be overtly emphasized [2, 8, 11]. A few authors have however clearly documented and discussed neonatal case-specific fatality rates. For instance, Owa et al. [12] in a 10-year retrospective review of neonatal admissions in a tertiary hospital in Southwestern Nigeria over two decades ago, calculated and documented the case fatality rates for common neonatal conditions that were responsible for neonatal mortality. Case-specific fatality rates were also computed and discussed by Zuniga [13] et al. in Burundi as well as Kotwal et [14] al in India. The focus of most community-based neonatal studies is the determination of sociodemographic risk factors for neonatal mortality [15–19]. On the other hand, hospital-based studies have the potential to identify specific causes of neonatal mortality [5, 20–22]. These two types of research studies complement each other in providing comprehensive data for holistic neonatal interventions. There is no doubt that neonatal case-specific fatality rate as an index for evaluating neonatal care has not been given adequate attention in Nigeria, thus justifying the need for the current hospital-based study conceived to determine the case-specific mortality rates for major causes of neonatal mortality in our hospital.

## Methods

### Objectives of study

This hospital-based retrospective review was conducted to determine the causes of neonatal mortality as well as compute case-specific fatality rates for the most prevalent morbidities at the National Hospital Abuja to highlight case-specific fatality rate as an important neonatal health index. It was hoped that the findings would trigger a paradigm shift in neonatal care practice and hopefully bring about significant reduction in neonatal mortality rate in Nigeria.

### Study site

The study was conducted in a 500-bed tertiary hospital located in the Federal Capital Territory, which provides care for residents of the city and patients from six adjoining states. The hospital operates a level 2 neonatal intensive care unit with an admission capacity of 45 babies and an admission rate of 1500–2000 babies per annum with a slight preponderance of out born (55%) over inborn babies (45%). The out born babies are usually referred from other hospitals but in some cases are self-referred, for babies born at home. There are facilities for non-invasive respiratory support [bubble continuous positive airway pressure (BCPAP)], and all inborn very preterm babies are commenced on BCPAP as soon as they are stabilized in the labour room but surfactant administration is limited by parental affordability. The unit has a constant supply of medical oxygen and pressurized air, each from a central source. Caffeine citrate or aminophylline is used in very low birth weight babies to prevent apnea of prematurity and automated infusion pumps are available for administration of fluids and medications. Extreme preterm babies receive continuous breast milk drips via NGT powered by infusion pump devices and this is continued until they are able to tolerate intermittent bolus feeding.

For severely asphyxiated babies, the unit protocol focuses attention on fluid management, seizure control using phenobarbitone, mannitol infusion for raised intracranial pressure and provision of adequate calories. We do not have facilities for therapeutic cooling. Phototherapy is available for all babies admitted for jaundice and double volume exchange blood transfusion is carried out for babies with severe hyperbilirubinaemia (> 20 mg/dl for term babies, > 15 for preterm babies) The surgical department provides skilled Paediatric surgical support to the neonatal unit and the Obstetrics department conducts about 1000–1500 deliveries per annum with a Caesarean section (C/S) rate of 53%.

Data collection and analysis: Starting from January 2017, an electronic Neonatal Registry database using Research Electronic Data Capture (REDCap) software was introduced in our newborn unit in partnership with 2 other tertiary institutions in the country, funded by Indiana University, USA for the systematic collection of Patient and Centre level Clinical and Outcome Data. The objective of this was to provide high quality data to support clinical research, quality improvement efforts and stimulate collaborative efforts. Further details have been provided in an earlier publication [23]. This is the second of a two–part preliminary analysis of NICU admissions following the introduction of the Neonatal Registry. Data for this study was retrospectively collected on all neonatal admissions at the NHA over a period of 13 months (January 2017 to February 2018). We retrieved relevant demographic and clinical data from the Neonatal Registry database, (REDCap). These included gestational age (GA). Birth weight (BW), place of delivery (POD), mode of delivery (MOD), maternal parity, antenatal care, admission diagnosis, final diagnosis and postnatal (hospital) care. For the purpose of analysis, the final/discharge diagnosis was used where this differed from the admission diagnosis. Where a baby was managed for more than one clinical condition, the documented perceived ‘more significant’ morbidity was used to categorize the baby. If this was not possible, the baby was excluded from analysis. Severe perinatal asphyxia was diagnosed when Apgar score was ≤3 at 5 min in addition to signs of acute brain dysfunction (seizures, unconsciousness, tone abnormality, bulging anterior fontanel) and for out born babies without a clear record/documentation of Apgar scores, a history of poor cry at birth in addition to clinical evidence of acute brain dysfunction was used. The diagnosis of RDS was as previously described [23] while major congenital malformations were defined as gross structural defects of body or organs present at birth capable of impairing viability and therefore requiring intervention [24]. Babies were classified as neonatal sepsis in the presence of suspicious clinical signs and positive blood culture.

### Data analysis

Data was analyzed using the Statistical Package for Social Sciences SPSS version 20 (IBM SPSS Armonk, NY). Overall mortality as well as the mortality rates for specific morbidities were computed and the percentage contribution to overall mortality of all identified illnesses was determined. For the purpose of analysis, ‘mortality’ was limited to deaths occurring in hospital within the first 28 postnatal days.

We went further to calculate the number of deaths resulting from specific conditions as a fraction of total number admitted for each of the 5 most common morbidities (number of death from a disease/total number of babies admitted with the disease) to arrive at the case-fatality rates. Using the number of deaths among inborn admissions and total number of deliveries in the hospital during the period of study, the facility-level neonatal mortality rate was also computed. Risk factors for neonatal mortality were identified using Odds Ratio with 95% confidence interval and the difference in the pattern of distribution of causes of mortality among inborn and out born babies was assessed using Chi square statistics. A significant *P* value was set at < 0.05.

## Results

As shown in Table 1, there were 730 babies with a preponderance of males 391(53.9%), Caesarian section deliveries 384(53.3%), inborn 396(54.5%) and term 396(54.2%) babies. Thirty-seven (4.8%) were extreme preterm, 40 (6.4%) were extreme low birth weight (birth weight < 1000 g) while 35 (5.2%) were macrosomic (birth weight ≥ 4000 g) and 715 (97.9%) of the mothers had some level of education, 393 (58%) were in the low multiparity group and 82 (11.2%) received antenatal corticosteroid. Gestational age estimation was by Last Menstrual Period in 704 (96.4%) mothers.

**Table 1:** General/Social demographic characteristics

General characteristics (n)	Frequency (%)
Sex distribution: (726)	
male	391(53.9)
female	335(46.1)
Mode of delivery: (720)	
spontaneous vertex delivery	336 (46.7)
elective caesarean section	290 (40.3)
emergency caesarean section	94 (13.0)
Place of delivery: (727)	
inborn	396 (54.4)
out born (hospital)	299 (41.4)
out born (home)	32 (4.4)
Gestational age category: (730)	
< 28 weeks	37 (4.8)
28–33 weeks	181 (24.8)
34–36 weeks	116 (15.4)
≥ 37 weeks	396 (54.2)
Birth weight category: (669)	
Extreme Low Birth Weight (< 1000 g)	40 (6.4)
Very Low Birth Weight (1000-1499 g)	100 (15.0)
Low Birth Weight (1500–2.499 g)	178 (26.6)
Normal Birth Weight (2500-3999 g)	316 (47.2)
Macrosomia (≥4000 g)	35 (5.2)
Maternal educational status: (730)	
Nil	15 (2.1)
Primary	39 (5.3)
Secondary	190 (26.0)
Tertiary	438 (60.0)
Unknown	48 (6.6)
Parity of mother: (730)	
Nulliparity (0)	275 (37.7)
Low parity (1–3)	393 (58.3)
High parity (≥4)	62 (8.5)
Antenatal corticosteroid: (730)	
Yes	82 (11.2)
No	635 (87.0)
Not known	13 (1.8)
Method of gestational age estimation (730)	
Last Menstrual Period	704 (96.4)
1st Trimester Ultrasound	2 (0.3)
Ballard	64 (8.9)
Continuous Positive Airway Pressure use (730)	
Yes	188 (25.8)
No	542 (74.2)

The most commonly encountered morbidities were prematurity related complications (36.7%), neonatal jaundice (28.1%), perinatal asphyxia (12.3%), congenital malformations (11.2%) and neonatal sepsis (8.8%). One hundred and thirteen babies died giving an overall mortality of 15.5% (prematurity 6.4%, congenital malformations 3.7%, perinatal asphyxia 3.6%, sepsis 1.1%, neonatal jaundice 0.3% and others 0.4%). Mortality rates for inborn and out born babies were 11.4 and 17.8% respectively and the difference was statistically significant, (*P* = 0.013). The total number of deliveries (life births) in our hospital during the period of study was 1635, out of which 396 (24%) required NICU care and 44 neonatal deaths (inborn) were documented among them giving an in-hospital neonatal mortality rate (NMR) of 26.9 per 1000 live births.

**Fig. 1:**
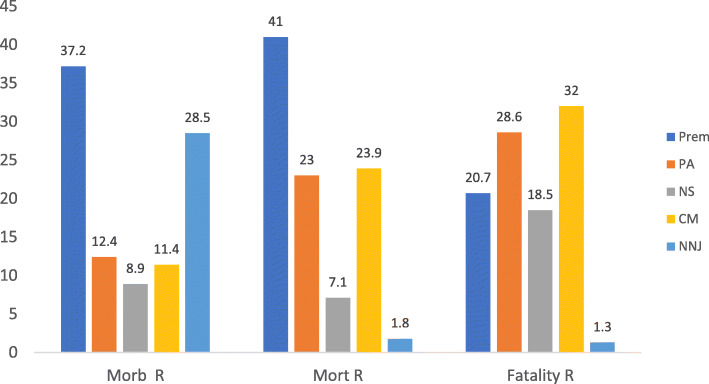
Morbidity, mortality and case specific fatality distribution of 5 top causes of neonatal admissions. Prem = Prematurity. PA = Perinatal asphyxia. NS = neonatal sepsis. CM = Congenital malformations. NNJ = Neonatal jaundice

Figure 1 shows the proportional contribution of 5 major morbidities to overall mortality as well as their case-specific fatality rates. Prematurity (37.2%) and neonatal jaundice (28.4%) contributed the highest proportions of morbidity and prematurity was responsible for the highest proportion (41.6%) of mortality but congenital malformations (32.5%) and perinatal asphyxia (28.6%) were associated with the highest case-specific fatality rates among the babies. The 3 top causes of mortality were prematurity (41.6%), congenital malformations (23.9%) and Perinatal Asphyxia (23.0%).

Table 2 shows the causes of neonatal mortality in relation to place of birth. There is a significant difference in the distribution of the major causes of death among inborn and out born babies in our study, (X^2^ = 21.669, *P* = 0002). While prematurity was the leading cause of death among inborn babies, congenital malformation was the predominant cause of mortality among out born babies. Although perinatal asphyxia and neonatal sepsis were responsible for higher proportions of deaths among out born babies (27.6 and 8.6% respectively) compared to inborn babies (17.8 and 6.7% respectively), the differences were not statistically significant. (*P* = 0.18 and 0.94respectively).

**Table 2:** Distribution of major causes of neonatal mortality among inborn and out born babies

Morbidity	Contribution to neonatal mortality				
	Inborn (%)	Out born (%)	Total (%)	X^2^	*P*
Prematurity	30(68.2)	13(24.1)	43(43.9)	19.954	0.0001
Perinatal Asphyxia	8(18.2)	16(29.6)	24(24.5)	1.718	0.18
Cong. Malformation	03(6.8)	20(37.0)	23(23.5)	12.327	0.0005
Neonatal Sepsis	03(6.8)	05(9.3)	08(8.2)	0.005	0.94
Total	44 (100.0)	54(100.0)	98(100.1) **X**		
X^2^ = 21.669		*P* = 0.0002			

**X:** total number less than 113 because specific information about place of delivery was missing in some patients. % represent column percentages

As shown in Table 3, 14 (51.9%) of the fatal congenital malformations were in the gastrointestinal system, 6 (22.2%) were non-specified multiple congenital malformations while the others were in the central nervous system 3 (11.1%), urogenital system 3 (11.1%) and cardiovascular system 1 (3.7%). Twenty (87%) of the fatal congenital malformations were out born babies referred from other hospitals while only 2 (13%) were inborn.

**Table 3:** Systemic classification of fatal congenital malformations

System involved (n)	Specific malformation	Frequency (%)
Gastrointestinal system (14)	Oesophageal atresia	5 (18.5)
	Ruptured Omphalocele major	2 (7.4)
	Malrotation with volvulus	2 (7.4)
	Caecal perforation	1 (3.7)
	Ladds band	1 (3.7)
	Ileal atresia	1 (3.7)
	Gastroschisis	1 (3.7)
	Jejunal atresia	1 (3.7)
Central nervous system (3)	Congenital hydrocephalus:	
	• Arnold Chiari malformation • Dandy Walker syndrome Ventriculomegaly	1 (3.7) 1 (3.7) 1 (3.7)
Urogenital system (3)	Posterior urethral valve	3 (11.1)
Multiple congenital malformations (6)	Non specified	6 (22.2)
Cardiac (1)	Cyanotic Congenital heart disease	1(3.7)

The risk factors associated with neonatal mortality are explored in Table 4. Gender was not a significant risk factor for mortality in this study however, place of delivery, gestational age, mode of delivery and birth weight category were significantly associated with neonatal mortality (OR, 95%CI shown in Table 4). Out born babies were more likely to die than their inborn counterparts and babies delivered by caesarean section had a much lower mortality rate than those delivered spontaneously per vagina.

**Table 4:** Risk factors associated with neonatal mortality

Variable	Mortality rate (%)	COR	CI
Place of delivery			
Out born	17.8	1.69	1.11, 2.55
Inborn	11.4		
Mode of delivery			
spontaneous vertex delivery	23.0	3.54	2.14, 5.86
caesarean section	5.7		
Gender			
Male	13.8	0.82	0.54, 1.23
Female	16.4		
Gestation			
Preterm	20.7	2.03	1.35, 3.05
Term	11.3		
Birth weight category			
< 2500 g	22.3	4.12	2.37, 7.17
≥ 2500	5.4		

## Discussion

This study reveals a pattern of neonatal morbidity in Abuja characterized by preponderance of preventable neonatal illnesses (neonatal jaundice, perinatal asphyxia and prematurity related complications.) which differs from the pattern previously reported from different parts of Nigeria where neonatal sepsis had consistently been among the 3 most common causes of NICU admissions [2–4, 7, 25, 26]. Furthermore, the significant contribution of major congenital malformations to neonatal mortality in this study, uniquely deviates from findings in previously conducted studies which had reported perinatal asphyxia, neonatal sepsis and prematurity as the three top causes of neonatal mortality [3, 27]. The low prevalence of neonatal sepsis may be due to our strict definition of sepsis (suspicious clinical signs + positive blood culture), while the unusual contribution of congenital malformations to neonatal mortality could be attributed to referral of complex congenital malformations to our hospital, the only tertiary referral center in this region with the capacity to treat complex surgical conditions. High level of maternal education might contribute to the low incidence of sepsis because of the higher tendency to understand and adhere to infection prevention habits among educated mothers. Furthermore, the heightened level of awareness among educated mothers may positively influence their health seeking behaviour and this may partly explain the large number of major congenital abnormalities seen in this study. The positive impact of maternal education on child mortality was highlighted by Fei Yu et al. [28].

Fatal congenital malformations reported in our study were predominantly seen among out born babies, and the poor outcome probably resulted from late arrival in the hospital, when complications capable of jeopardizing post-operative survival had set in. Mmbaga et al. [ 29] in Tanzania reported a mortality pattern with some similarity to our findings; perinatal asphyxia (45.7%), prematurity (35.1%) and congenital malformations (9.0%) being the three leading causes of death in their neonatal unit. The report was however limited to inborn babies and this may explain the lower contribution of congenital malformations to neonatal mortality relative to our findings. Fatal congenital malformations in our study were predominantly seen among out born babies.

The mortality rate of 15.5% falls within the range of 14.2–20.4% previously reported from different parts of Nigeria [2, 4, 8, 11, 25, 30, 31]. This lends credence to the National Demographic and Health Survey (NDHS) report that neonatal mortality rate (NMR) had not changed significantly in Nigeria in the last two decades [8]. It is worthy of note that mortality rate in this study was significantly higher among out born babies compared to inborn babies and this finding was similarly reported in some previous studies in Nigeria (26.8% Out born versus 10.9% inborn in Calabar [30], 20.5% out born versus 6.4% inborn in Kano [4]). Sick out born babies have no access to effective resuscitation at the birthing facility, receive poor neonatal care during transportation and arrive late to the referral hospital, these factors synergistically culminate in poor outcome.

Congenital malformations and Perinatal asphyxia had the highest neonatal case-specific fatality rates in our study (32.5 and 28.6% respectively). This is in sharp contrast to case-specific fatality rates previously reported from Nigeria by Omoigberale et al. [2] (1 and 33.4% for congenital malformations and Perinatal Asphyxia respectively) and from Burundi; 10 and 18% for congenital malformation and perinatal asphyxia respectively) [ 13]. Although not stated in their study, we speculate that Omoigberale et al. [2] admitted babies with low-risk congenital malformations in their unit and this may be due to low level of awareness about available care for major congenital malformations at that time. The cases in our study were high-risk congenital malformations, predominantly referred from other hospitals, with complications at presentation. These complications precluded surgical intervention in some patients while jeopardizing post-operative survival in others. For instance, the omphaloceles were ruptured and potentially infected, gastroschisis was associated with intestinal ischaemic gangrene while babies with oesophageal atresia had developed aspiration pneumonitis. In the absence of facilities and surgical skills to manage babies with major congenital malformations at the lower-level hospitals, the babies were deliberately referred to higher level hospitals, resulting in an apparently low contribution to neonatal mortality at the referring hospital as was reported by Garba et al. [31] and high fatality rate at the receiving hospital. A high fatality rate (62.2%) for surgical congenital malformations was reported from a tertiary hospital in Uyo, southern Nigeria, and most of the patients were referred from other hospitals with complications on arrival [30]. The pattern and outcome of congenital malformations in their report was similar to that of our study with a preponderance of gastrointestinal malformations.

The risk of death (fatality) from prematurity related morbidities (17.3%) was lower than that of perinatal asphyxia and congenital malformations. Increased survivability of preterm babies in our center in the last few years may be due to improvement in neonatal care (utilization of CPAP for respiratory distress syndrome, institutionalization of Kangaroo mother care, use of automated infusion pumps for administration of fluid and medications, breast milk drip for continuous enteral feeding through nasogastric tubes). Neonatal sepsis accounted for only 7% of all admissions and its fatality rate was 12.3%. The low fatality rate is attributable to the use of specific antibiotics from the sensitivity test result; all were bacteriologically confirmed and antibiogram was available in every case.

Death from congenital malformation was more prevalent among out born babies compared to their inborn counterparts. Mortality from perinatal asphyxia was also more prevalent among out born babies although the difference was not statistically significant. This is similar to the pattern reported from the Southern part of Nigeria [30]. The apparent survival advantage of the inborn asphyxiated baby over the out born asphyxiated baby may be due to inadequate resuscitation at birth as well as delayed access of the out born babies to post asphyxia care. Regular training and retraining of staff on newborn resuscitation will be more impactful in the presence of a collaboration between different levels of health care service delivery that enables constant supervision of the lower-level centers by the higher-level hospitals.

The present study showed that birth weight, gestational age, place of delivery and mode of delivery were significantly associated with mortality. Inborn, term and high birth weight babies as well as those delivered by caesarian section were more likely to survive than out born, preterm and low birth weight babies delivered per vagina. The harsh conditions which sick out born babies are exposed to in the process of transfer from one hospital to another would necessarily impact negatively on their outcome. Similar findings had been reported by other authors; Bello et al. [32] reported a higher risk of death among out born preterm babies of lower gestation in Maiduguri, North Eastern Nigeria while Nga et al. [18] identified poor referral system, place of birth and ethnicity as risk factors for neonatal deaths in rural north Vietnam.

Over 75% of abdominal deliveries among our patients were performed presumably as a salvage procedure in most of the babies and this may account for the lower risk of mortality associated with caesarean section delivery. A higher proportion of early neonatal deaths resulting from perinatal asphyxia in Brazil were seen among vaginal deliveries and this was attributed to poor monitoring in labour and delivery which did not allow for identification of at-risk babies for emergency abdominal delivery [33].

### Limitation

Some errors may have occurred in the assignment of cause of death in some cases of multiple morbidities. We however believe that the number of cases in this category was minimal and the overall effect on the analysis negligible.

## Conclusions

Major congenital malformations and perinatal asphyxia are associated with the highest case-specific neonatal mortality at the National hospital Abuja and this is not unconnected with out born delivery and delayed arrival of referred babies to the hospital. Early antenatal diagnosis of congenital malformations through mandatory antenatal anomaly scan is recommended. Furthermore, we recommend perinatal identification of babies at risk for asphyxia at the lower level hospitals, for immediate in-utero transfer to a tertiary health facility for delivery. There is an urgent need for a functional network system between different levels of hospitals in Nigeria to facilitate inter-hospital patient referral for optimal neonatal care and intensification of training of all staff responsible for the immediate care of the newborn in all facilities where deliveries are conducted including traditional birth attendants who conduct deliveries outside the hospitals.

## Data Availability

The datasets analysed for this current study are available from the corresponding author on reasonable request.

## Abbreviations

BCPAP: Bubble Continuous Positive Airway Pressure
BW: Birth weight
GA: Gestational age
MOD: Mode of delivery
NHA: National Hospital Abuja
NGT: Naso-gastric tube
NNS: Neonatal sepsis
NDHS: National Demographic and Health Survey
NMR: Neonatal Mortality Rate
PA: Perinatal Asphyxia
REDCap: Research Electronic Data Capture

## References

[1] Eke CB, Ezomike UO, Chukwu BF, Chinawa JM, Korie FC, Chukwudi N, Ukpabi IK (2014). Pattern of neonatal mortality in a tertiary health Facilityin Umuahia, south eastern Nigeria. Int J Trop Dis Health.

[2] Omoigberale AI, Sadoh WE, Nwaneri DU (2010). A 4 year review of neonatal outcome at the University of Benin teaching hospital, Benin City. N J Clin Pract.

[3] Okechukwu AA, Achonwa A (2009). Morbidity and mortality patterns of admissions into the special care baby unit of University of Abuja Teaching Hospital, Gwagwalada, Nigeria. N J Clin Pract.

[4] Mukhtar-Yola M, Iliyasu Z (2007). A review of neonatal morbidity and mortality in Aminu Kano teaching hospital, northern Nigeria. Trop Dr.

[5] Guerrier G, Oluyide B, Keramarou M, Grais R (2013). High maternal and neonatal mortality rates in northern Nigeria: an 8-month observational study. Int J Women's Health.

[6] Fottrell E, Osrin D, Alcock G, Kishwar A, Ujwala B, James B (2015). Cause-specific neonatal mortality: analysis of 3772 neonatal deaths in Nepal, Bangladesh, Malawi and India. Arch Dis Child Fetal Neonatal Ed.

[7] National Population Commission (NPC) [Nigeria] and ICF (2019). Nigeria demographic and health survey 2018 key indicators report.

[8] Abdullahi UI (2018). Neonatal morbidity and mortality in a rural tertiary Hospital in Nigeria. CHRISMED J Health Res.

[9] Ramadurg UY, Ghattargi CH, Gagan S, Manjula R, Mayappanavar RY, Bhadja D, Nair SS (2014). A study of causes of neonatal mortality in tertiary care hospital, Bagalkot. Intern J Health Inf Med Res.

[10] Kanchan T, Kumar N, Unnikrishnan B, Payne-Jane J, Byard RW (2016). Mortality: Statistics. Encyclopedia of Forensic and Legal Medicine.

[11] Ekwochi U, Ndu IK, Nwokoye IC, Ezenwosu OU, Amadi OF, Osuorah D (2014). Pattern of morbidity and mortality of newborns admitted into the sick and special care baby unit of Enugu State University teaching hospital, Enugu state. Niger J Clin Pract.

[12] Owa JA, Osinaike AI (1998). Neonatal morbidity and mortality in Nigeria. Indian J Pediatr.

[13] Zuniga R, Van den Bergh B, Ndelema D, Bulckaert M, Manzi V, Lambert R (2013). Characteristics and mortality of neonates in an emergency obstetric and neonatal care facility, rural Burundi. Public Health Action.

[14] Kotwal YS, Yatoo GH, Ahmed Jan FA (2017). Morbidity and mortality among neonates admitted to a neonatal intensive care unit of a tertiary care teaching Hospital of Jammu and Kashmir (India). Neonat Pediatr Med.

[15] Ezeh OK, Agho KE, Dibley MJ, Hall J, Page AN (2014). Determinants of neonatal mortality in Nigeria: evidence from the 2008 demographic and health survey. BMC Public Health.

[16] Morakinyo OM, Fagbamigbe AF (2017). Neonatal infant and under-five mortalities in Nigeria: an example of trends and drivers (2003-2013). PLoS One.

[17] Akinyemi OJ, Bamgboye EA, Ayeni O (2015). Trends in neonatal mortality in Nigeria and effects of bio-demographic and maternal characteristics. BMC Pediatr.

[18] Wolde HF, Gonete KA, Akalu TY, Baraki AG, Lakew AM (2019). Factors affecting neonatal mortality in the general population: evidence from the 2016 Ethiopian demographic and health survey EDHS0-multi level analysis. BMC Res Note.

[19] Nga NT, Hoa DT, Malquist M, Persson LA, Ewald U (2012). Causes of neonatal deaths: results from NeoKIP community-based trial in Quang Ninh Province, Vietnam. Acta Paediatr.

[20] Moise IK (2018). Causes of morbidity and mortality among neonates and children in post-conflict Burundi: a cross-sectional retrospective study. Children.

[21] Garg VK, Singh MN, Mishra OP, Gupta R, Bhatia BD, Bhargava V (1987). Neonatal mortality rate: a hospital study. Indian Pediatr.

[22] Rajab AM, Ghareba AM (2013). Neonatal mortality rate in the special care baby unit (SCBU) at Gharian teaching hospital. J Med Sci Clin Res.

[23] Audu LI, Otuneye AT, Mairami AB, Mukhtar-Yola M, Mshelia LJ, Ekhaguere A (2020). Gestational age-related neonatal survival at a tertiary health institution in Nigeria: The age of fetal viability dilemma. Niger J Paediatr.

[24] Singh K, Krishnamurthy K, Greaves C, Kandamaran L, Nielsen AL, Kumar A. Major congenital malformations in Barbados: the prevalence, the pattern, and the resulting morbidity and mortality ISRN obstetrics and gynecology. Obstet Gynaecol. 2014:8. 10.1155/2014/651783 Article ID 651783. 10.1155/2014/651783 PMC4003834 25006483

[25] Ezechukwu CC, Ugochukwu EF, Egbuonu I, Chukwuka JO (2004). Risk factors for neonatal mortality in a regional tertiary Hospital in Nigeria. Niger J Clin Pract.

[26] Adetola AO, Tongo OO, Orimadegun AE, Osinusi K (2011). Neonatal mortality in an urban population in Ibadan, Nigeria. Paediatr Neonatol.

[27] Also U, Gwarzo GD (2020). Patterns of morbidity and mortality among neonates seen in a tertiary hospital. Sahel Med J.

[28] Yu F, Yan Z, Pu R, Tang S, Ghose B, Huang R. Mothers with lower socioeconomic status contribute to the rate of all-cause child mortality in Kazakhstan. Biomed Res Int. 2018:8 http://doi.org/101155/2018/3629109. Article ID3629109. 10.1155/2018/3629109 PMC5832164 29651427

[29] Mmbaga BT, Lie RT, Olomi R, Mahande MJ, Kvåle G, Daltveit AK (2012). Cause-specific neonatal mortality in a neonatal care unit in northern Tanzania: a registry-based cohort study. BMC Pediatr.

[30] Udo JJ, Anah MU, Ochigbo SO, Etuk I, Ekanem AD (2008). Neonatal morbidity and mortality in Calabar, Nigeria: a hospital-based study. Niger J Clin Pract.

[31] Garba BI, Muhammad AS, Mohammed BA, Obasi AB, Adeniji AO (2017). A study of neonatal mortality in a specialist hospital in Gusau, Zamfara, North-Western Nigeria. Int J Trop Dis Health.

[32] Bello M, Pius S, Ibrahim BA (2019). Characteristics and prediction of outcome of care of preterm newborns in resource constrained setting, Maiduguri, north eastern Nigeria. J Clin Neonatol.

[33] Daripa M, Caldas HM, Flores LP, Waldvogel BC, Guinsburg R, de Almeida MF (2013). Perinatal asphyxia associated with early neonatal mortality: populational study of avoidable deaths. Rev Paul Pediatr.

